# Development and long-term evaluation of a new ^68^Ge/^68^Ga generator based on nano-SnO_2_ for PET imaging

**DOI:** 10.1038/s41598-020-69659-8

**Published:** 2020-07-29

**Authors:** Eduardo Romero, Alfonso Martínez, Marta Oteo, Marta Ibañez, Mirentxu Santos, Miguel Ángel Morcillo

**Affiliations:** 1 Biomedical Applications and Pharmacokinetics Unit, CIEMAT, 28040 Madrid, Spain; 2 Molecular Oncology Unit, CIEMAT, 28040 Madrid, Spain

**Keywords:** Preclinical research, Nuclear chemistry

## Abstract

Radionuclide generator systems can routinely provide radionuclides on demand such as ^68^Ga produced by a ^68^Ge/^68^Ga generator without the availability of an on-site accelerator or a research reactor. Thus, in this work nano-SnO_2_ was used to develop a new ^68^Ge/^68^Ga generator which was evaluated over a period of 17 months and 305 elution cycles. The elution yield was 91.1 ± 1.8% in the first 7 mL (1 M HCl as eluent) when the generator was new and then it decreased with time and use to 73.8 ± 1.9%. Around 80% of the elutable ^68^Ga activity was obtained in 1 mL and the ^68^Ge content in the eluate did not exceed 1 × 10^–4^% over the investigation period when it was eluted regularly. The described generator provided adequate results for radiolabelling of DOTA-TOC with direct use of eluate. In addition, [^68^Ga]Ga-DOTA-TOC was tested satisfactorily for in vivo tumor detection by microPET/CT imaging in a lung cancer mouse model.

## Introduction

^68^Ga-based radiopharmaceuticals are very relevant to Positron Emission Tomography (PET) because this radionuclide has decay characteristics suitable for PET imaging^1^. ^68^Ga disintegrates to ^68^Zn by positron emission (88.88%) with a maximum energy of 1899.1 keV and partially by electron capture (11.11%), and has a physical half-life (T_1/2_) of 67.845 ± 0.018 min (mean ± variance)^1^, pharmacokinetically compatible with a number of molecules with a high clearance such as those of low molecular weight, antibody fragments and peptides. Due to the fact the ^68^Ga can form stable complexes with many ligands containing donor atoms such as nitrogen and oxygen means that a great variety of chelating agents can be used for complexation. ^68^Ga can also form macromolecules useful in the diagnosis, treatment and follow-up of patient response to chemo- and radiotherapy^2, 3^.

Gallium-68 is the product of the radioactive decay of ^68^Ge and can be obtained by using a ^68^Ge/^68^Ga generator. Germanium-68 has a T_1/2_ = 270.95 ± 0.26 days^4^, and this permits the manufacture of potentially inexpensive and long-lived generator systems^5^. This is an advantage over usual PET radionuclides produced via proton irradiation such as ^18^F, ^11^C, ^13^N, ^15^O, ^64^Cu and ^124^I. An additional benefit of using ^68^Ga is that it can be separated easily on demand and its availability in highly-specific activity, no-carrier-added form^6, 7^. Furthermore, the higher resolution of PET when compared to Single Photon Emission Computed Tomography (SPECT) could result someday in ^99m^Tc or ^111^In-based radiopharmaceuticals being replaced by those based on ^68^Ga.

The most common commercially available ^68^Ge/^68^Ga radionuclide generators nowadays are discussed here. Despite remarkable advancements, the content of ^68^Ge in the eluate (breakthrough) and metallic impurity presence in generator eluate appear to be the greatest impediments to direct-labelling of ^68^Ga in the manufacture of ^68^Ga-based radiopharmaceuticals. Potential metal ions from generator eluate may challenge ^68^Ga^3+^ for coordinative labelling of radiopharmaceutical precursors and therefore produce lower ^68^Ga incorporation or a reduction of specific radioactivity in the ultimate ^68^Ga-labelled radiopharmaceutical^3^. The European Pharmacopoeia^8^ specifies that gallium (^68^Ga) chloride solution shall not contain more than 10 µg of Fe and Zn per GBq of ^68^Ga in eluate that is used for radiolabelling and that no more than 0.001% of ^68^Ge should be present in the ^68^Ga eluate. Table 1 gives an outline of the characteristics of major commercial ^68^Ge/^68^Ga generators currently available on the market. Two of the most commonly-used, commercially-available ^68^Ge/^68^Ga generators are TiO_2_ and SnO_2_ generators. Cyclotron Co, Ltd. (Obninsk, Russian Federation) has developed a generator based on a modified TiO_2_ phase support. This generator is eluted with 0.1 M HCl and shows a ^68^Ga elution yield > 80% with a ^68^Ge breakthrough not more than 5 × 10^–3^% when the generator is fresh. The ^68^Ga elution yield decreases over time (in 3 years or after 400 elutions) to around 45% and the ^68^Ge breakthrough levels per elution increase to about 10^–2^% over extended periods of generator usage^9–11^. The metal ion content in the eluate is not more than 2 ppm for some metals (Pt, Ba, Fe, Zn, Mn, Pb, Ti, Cr, Al, Cd, Co, Cu, Ni and V)^10, 11^. Eckert & Ziegler (Germany) manufacture a similar TiO_2_-based generator which provides improved elution characteristics and has been approved for clinical use under the brand name of ‘GalliaPharm®’ in Europe and the United States. This system is eluted with 7 mL of 0.1 M HCl with a ^68^Ga elution efficiency of about 70% when the generator is new; this value can drop somewhat if the generator is not eluted for a few days. For this generator ^68^Ge breakthrough ranges typically from 3 × 10^–5^% when fresh to 5 × 10^–3^% upon repeated elutions (e.g. 200) or over time (e.g. after about 1 year); this value can go up to 1.0 × 10^–3^% if the generator is not eluted for a few days. After a break in use, the generator should be pre-eluted 1 day prior to its next intended use^7, 12^. Metallic impurities in the ^68^Ga eluate vary between 1 and 10 ppm for Cu, Fe, Al, Mn and Ti ions^13^.

**Table 1:** Characteristics of the ^68^Ge/^68^Ga generators currently available on the market.

Company	Column matrix	Eluent	Initial ^68^Ga elution yield (%)	^68^Ge breakthrough (%)	Metallic impurities (ppm)
Cyclotron Co., Ltd (Obninsk)	TiO_2_	0.1 M HCl	> 80	< 5 × 10^–3^	< 2
Eckert & Ziegler (GalliaPharm®)	TiO_2_	0.1 M HCl	> 70	3–5 × 10^–3^	1–10
iThemba LABS	SnO_2_	0.6 M HCl	> 80	< 2 × 10^–3^	1–20
ITG GmbH	Pyrogallol-derivatized SiO_2_	0.05 M HCl	> 80	< 5 × 10^–3^	< 0.2
Pars Isotope (PARS-GalluGEN®)	SnO_2_	0.6 M HCl	> 50	< 2 × 10^–5^	< 1
IRE EliT (Galli Eo®)	Unspecified	0.1 M HCl	> 65	< 1 × 10^–3^	< 1

iThemba LABS (Republic of South Africa) builds SnO_2_-based solid phase generator systems; this generator is eluted with 0.6 M HCl and has a yield of about 80% in 5 mL of eluent. ^68^Ge breakthrough for this generator is less than 2 × 10^–3^% at the reference date^7^. Total chemical impurities (as metal ions) are around 1–20 ppm for Sn, Fe, Cu, Mn, and Al^13^. Some other commercial generators are based on a modified dodecyl‐3,4,5‐trihydroxybenzoate hydrophobically bound to an octadecyl modified silica resin; ITG Isotope Technologies Garching GmbH (Munchen, Germany) produces this type of generator. ITG´s generator is eluted in 3–4 mL 0.05 M HCl with a ^68^Ge content 3 × 10^–3^–4 × 10^–4^% with an overall elution yield higher than 80%; ^68^Ge breakthrough decreases with use, which is a unique characteristic of this generator^14, 15^. Total metal impurities concentration is less than 0.2 ppm and ≤ 10 µg/GBq of ^68^Ga for Ni, Zn, Nb, Pb, Fe and Cu^13, 15^.

A number of new generators have recently entered the market. The first of these, which is based on SnO_2_ and known as ‘PARS-GalluGEN^®^, was developed by PARS Isotope (Tehran, Iran). When eluted with 0.6 M HCl, this generator has a ^68^Ga elution yield of about 50%^16^ and the ^68^Ge content in the eluate is less than 2 × 10^–5^%^17^. Fe, Sn and Zn concentrations in the eluate are less than 1 ppm^17^. Another emerging generator is Galli Eo (from IRE EliT, Fleurus, Belgium), however, the column contains an unspecified resin. The generator is eluted with 0.1 M HCl, and has more than 67% elution yield and less than 1 × 10^–3^% ^68^Ge breakthrough^18^. According to its brochure, metal content per elution is less than 1 ppm and ≤ 10 µg/GBq of ^68^Ga for Fe, Cu, Ni, Zn, Pb and Al. The Eckert & Ziegler GalliaPharm^®^ and the IRE ELiT Galli Eo^®^ generators are both GMP grade and have type II drug master files on file with the FDA.

The use of sorbents based on nanomaterials is an active area of research in the development of radionuclide generators. Over the past few years the use of nanoparticle-based sorbents has proven to be an interesting proposition due to their unique morphological features, pore structure, high surface areas and large numbers of surface active sites. This results in high sorption efficiency and selectivity in addition to significant chemical stability and radiation resistance compared to conventional sorbents^19^. Chakravarty et al*.* have developed a ^68^Ge/^68^Ga generator using a composite sorbent based on nanoceria-polyacrylonitrile (CeO2-PAN) for the preparation of their generator^20^. This generator is eluted with 0.1 M HCl and its elution profile is much sharper than the elution profiles of the commercial generators noted above; its elution yield for ^68^Ga is higher than 85% for the first few months of operation, gradually decreasing to about 70%. The breakthrough of ^68^Ge in the eluate is negligibly low (< 10^–4^%) and remains unchanged over the period of 1 year. Cu, Fe, Ce, Al, and Mn ion levels are always < 0.1 ppm^13–20^. This group has recently reported the clinical experience of 32 patients who were injected with [^68^Ga]-Ga-DOTA-NOC prepared using ^68^Ga eluted from the CeO_2_-PAN sorbent-based ^68^Ge/^68^Ga generator^21^. Our research group has also developed a nano-crystalline calcined SnO_2_-based ^68^Ge/^68^Ga generator. This in-house generator (1.85 MBq of ^68^Ge) has been evaluated over 100–200 elution cycles and shows markedly substantially high yield and reproducibility after repeated elutions^22^. This study will report on the development and long term performance evaluation of a novel generator with higher activity (740 MBq of ^68^Ge) based on this nano-metallic oxide, in terms of ^68^Ga elution yield, ^68^Ge breakthrough, and metallic impurities content of the eluate. Furthermore, we have demonstrated the suitability of the generator-eluted ^68^Ga for the radiolabelling of 1,4,7,10-tetraazacyclododecane-N,N′,N″,N‴-tetraacetic acid-d-Phe(1)-Tyr(3)-octreotide (DOTA-TOC) with the final aim of performing PET imaging studies in a preclinical mouse model of lung cancer.

## Results and discussion

### Operation of the ^68^Ge/^68^Ga radionuclide generator

**Figure 1:**
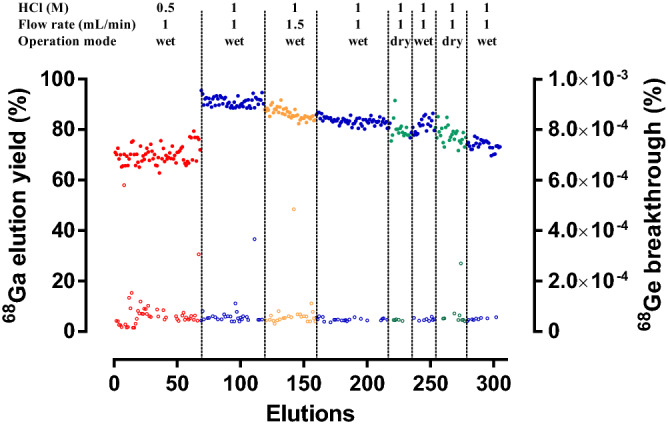
^68^Ga elution yield and ^68^Ge-breakthrough for the generator over 305 elutions. The generator was eluted with 7 mL of HCl under different elution conditions: (1) 0.5 M HCl; flow 1 mL/min; “wet” operation mode, (2) 1 M HCl; flow 1 mL/min; “wet” operation mode, (3) 1 M HCl; flow 1.5 mL/min; “wet” operation mode, and (4) 1 M HCl; flow 1 mL/min; “dry” operation mode. ^68^Ga elution yield and ^68^Ge-breakthrough are indicated as closed and open circles, respectively.

The ^68^Ge (740 MBq at calibration date) was completely adsorbed in the nano-SnO_2_ column and the ^68^Ge/^68^Ga generator was evaluated for preclinical research applications by examination of its performance over an extended period of 17 months (305 elutions). The generator was designed to operate either in the "dry" or "wet" operation mode (see “Materials and methods”). Over 70% of the ^68^Ga was eluted with 7 mL of 0.5–1 M HCl, as shown in Fig. 1. The elution yield of ^68^Ga was higher when eluted with 1 M HCl (91.1 ± 1.8%; n = 50), compared to elution with 0.5 M HCl (70.1 ± 3.5%; n = 68) when the generator was operated in the “wet” mode at 1 mL/min (Supplementary Table S1 online). Therefore, the generator was eluted using 1 M HCl from elution number 69 onward. This is in good agreement with previous results that described a ^68^Ge/^68^Ga generator based on SnO_2_ where the activities of ^68^Ga in the eluate increased when HCl concentration was raised from 0.5 to 10 M^23^. While elution with 2 M HCl could result in greater ^68^Ga activity in the eluate, as we have reported with an in-house built pilot ^68^Ge/^68^Ga generator based on nano-SnO_2_^22^, we preferred to elute the generator with lower acidity, at a maximum of 1 M, in order to preserve generator column integrity. ^68^Ga (1 M HCl, 1 mL/min) elution yield gradually decreased over time, ranging from 91.1 ± 1.8% (n = 50) to 73.8 ± 1.9% (n = 27) (Supplementary Table S1 online); these values are typical of ^68^Ge/^68^Ga generators based on inorganic oxides^7, 9–12, 24^. As was seen with the CeO-PAN-based ^68^Ge/^68^Ga generator, the decrease in elution yield over time was lower than was observed with commercial generators, in which the elution yield decreased to ~ 50% of its initial value^20^. Beyond the amount of eluted ^68^Ga, another great advantage of our generator is that its design permits operation in either "dry" or "wet" mode; no differences were found when the generator was operated in either mode (around 75–85%), thus confirming our previous findings^22^. An advantage of using HCl is that the environment it creates is hostile to microorganisms. This phenomenon has been reported when studying the survival of microorganisms such as fungal spores and acid-resistant bacteria in a generator column eluted with 0.1 M HCl. In this environment no viable microorganisms could be detected even over a prolonged testing period of several days. Risks associated with incidental microbial contaminations during the lifetime of such a generator were very low, as the conditions used in this type of generator were highly unfavourable for survival or growth of microorganisms^24^.

**Figure 2:**
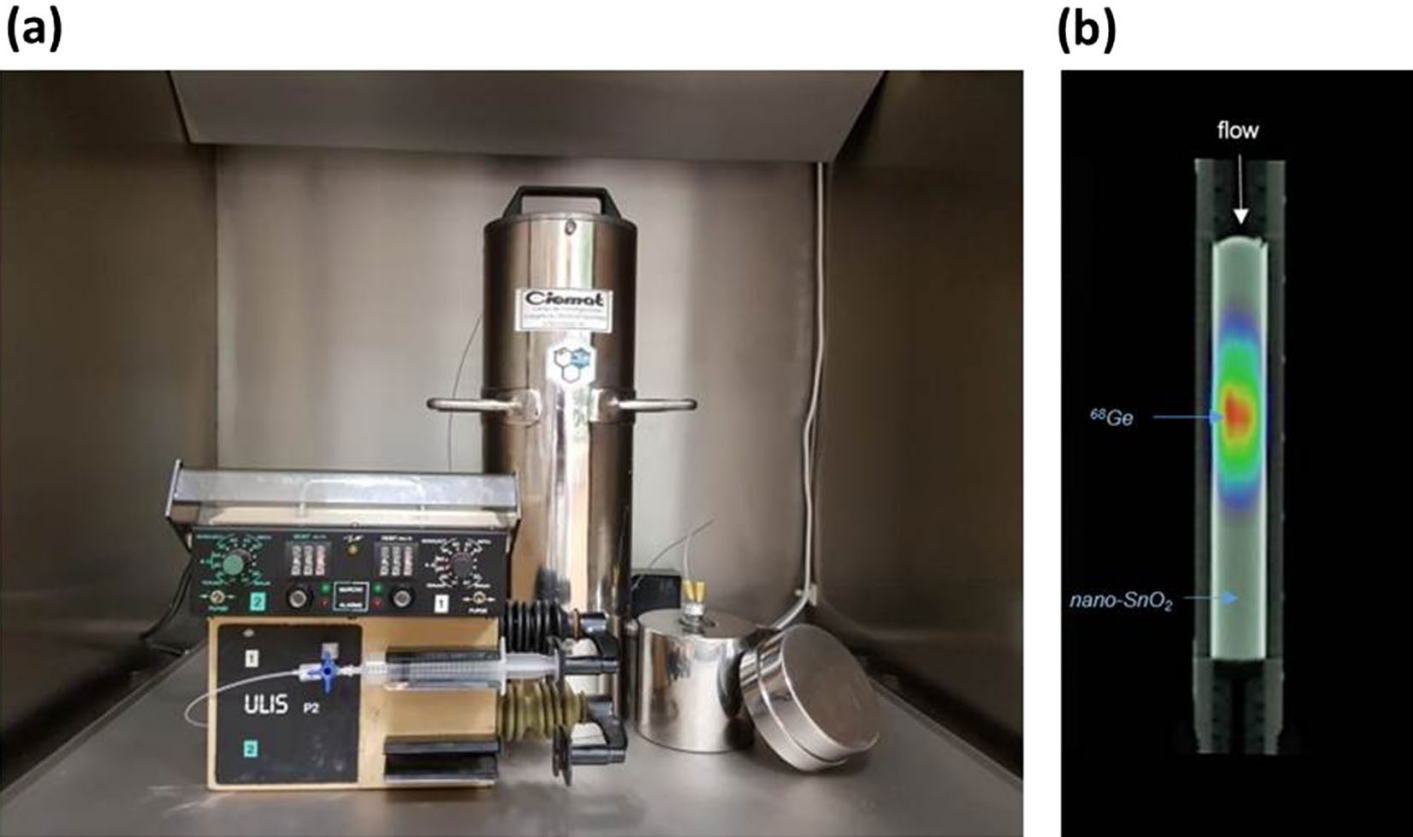
^68^Ge/^68^Ga generator. (**a**) The column was maintained in an easily-transportable lead shielded cylindrical shape. (**b**) Sagittal view PET/CT image of generator column after of 17 months (305 elution cycles).

Germanium-68 breakthrough was expressed as a percentage of ^68^Ge activity in the eluate with respect to ^68^Ge loaded at calibration time. ^68^Ge breakthrough from the generator was negligibly low (< 10^–4^%) and remained unchanged over the period studied (Fig. 1 and Supplementary Table S1 online). The generator’s quality in terms of ^68^Ge breakthrough was high, a result that has been confirmed in visualization by PET imaging of ^68^Ge adsorbed into the nano-SnO_2_-based sorbent. The PET image of the ^68^Ge/^68^Ga generator column after 305 elutions showed highest activity in the upper two-thirds of the column, i.e., no significant spreading and drifting of ^68^Ge zone in the column was observed after 17 months of use (Fig. 2). A slight increase in ^68^Ge breakthrough was observed on occasion when the generator was not eluted for more than 3 days, which is a phenomenon that had been observed in our previous study^22^. Nevertheless, after a few elutions, this always returned to values below 10^–4^%. For this reason daily elutions are recommended to keep the elution’s ^68^Ge content constant. We obtained the best results with the present nano-crystalline calcined SnO_2_-based ^68^Ge/^68^Ga generator (740 MBq of ^68^Ge) compared with our nano-SnO_2_-based in-house built pilot ^68^Ge/^68^Ga generator (1.85 MBq of ^68^Ge) which we had previously reported^22^. This can be explained by a greater amount of stationary phase used to prepare the generator with higher activity. The breakthrough obtained from our generator was considerably lower than with commercial ^68^Ge/^68^Ga generators^7, 9–14, 24^. These generators commercially supplied using SnO_2_ are based on the use of bulk tin oxide as sorbent and suffer a decrease in the elution yield of ^68^Ga and an increase of the breakthrough of ^68^Ge over a prolonged period of time^13^. An alternative is to use mesoporous metal oxides as sorbents for ^68^Ge/^68^Ga generators since they have demonstrated good performance and proven efficiency^25^. The average pore radius of our nano-SnO_2_-based ^68^Ge/^68^Ga generator was 14.8 nm indicating that the nano-crystalline SnO_2_ sorbent was mesoporous/macroporous. The mean crystal size was found to be 28.1 ± 0.5 nm; moreover, the particles of calcined SnO_2_ had a spherical shape with a high degree of agglomeration among fine particles^22^. The use of micron sized agglomerated particles is essential in the preparation of radionuclide generators since they decrease chemical impurities in the eluate^25^.

**Figure 3:**
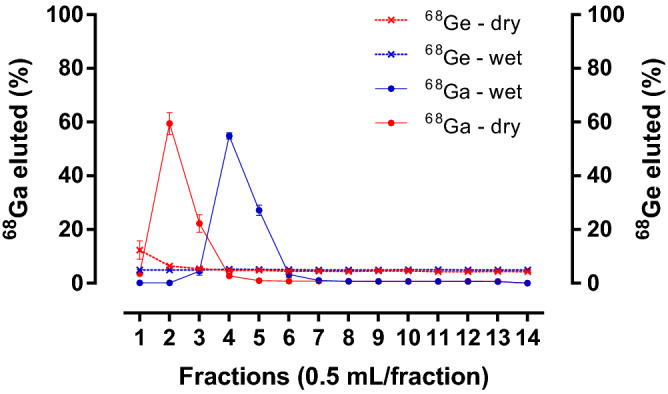
^68^Ga and ^68^Ge elution profiles. The generator was eluted with 7 ml of 1 M HCl at a flow rate of 1 mL/min operating in “dry” or “wet” mode. Fractions of 0.5 mL were collected. The data are expressed as mean ± standard deviation (n = 3).

Figure 3 shows the elution profiles of both radionuclides ^68^Ga and ^68^Ge, collecting fractions of 0.5 mL at a flow rate of 1 mL/min with 7 mL of 1 M HCl. The majority of ^68^Ga activity was eluted in fractions 2 and 3 when it was operated in “dry” mode (81.79 ± 1.00%; n = 3) and in fractions 4 and 5 when the generator was operated in “wet” mode (82.05 ± 1.12%; n = 3). While the percentage of the eluted ^68^Ge activity was very similar in all the fractions eluted in “wet” mode (5.01 ± 0.08%), a slight increase in the percentage of activity eluted in the first 1 mL (12.3 ± 3.4% and 6.5 ± 0.3% in the first and second fraction, respectively) was observed when the generator was operated in “dry” mode (Fig. 3). During the period studied the elution profile of both radionuclides remained unchanged. Therefore, if the generator is going to be operated in “wet” mode and its eluate will be used directly for ^68^Ga labelling procedures, the first 1.5 mL can be discarded and the subsequent 1 mL (containing the highest concentration of pure ^68^Ga) collected and buffered to an appropriate pH for radiolabelling. The elution profile obtained in “wet” mode was comparable to that reported in a detailed study of a commercial SnO_2_ generator^26^ and also with other commercial ^68^Ge/^68^Ga generators based on SnO_2_, TiO_2_, SiO_2_ and CeO_2_-PAN^13^.

Analysis of the selected decayed samples of ^68^Ga by Coupled Plasma-Mass Spectrometry (ICP-MS) revealed that levels of Ag, As, Ba, Bi, Ca, Cd, Co, Cr, Cu, Fe, In, Mg, Mn, Ni, Pb, Sb, Sn and Ti were always < 1 ppm, as shown in Table 2. The most frequently present impurity was Zn, primarily due to ^68^Ga decay and potential contamination in the preparation of 0.5–1 M HCl used to eluate the generator. In the ^68^Ge/^68^Ga generator column, the amount of Zn exceeds 10 times that of ^68^Ga at the time of secular equilibrium^27^, consequently, when the generator is eluted, if all the Zn in the column was eluted in 1 mL of 1 M HCl, the level of Zn would be negligibly low (< 0.1 ppm). “Dry” mode use of the ^68^Ge/^68^Ga generator resulted in Zn levels that were lower than when the “wet” mode was used (Table 2). When the generator was operated in “dry” mode, concentration Zn was below 0.5 ppm in half of the samples and in the other half, the mean concentration was 6.4 ± 7.9 ppm. Considerable care must therefore be taken during preparation of HCl solutions and samples for ICP-MS (zinc is a common contaminant in laboratories^28^). With regards to other metals, Fe and Cu ions are the most significant competitors with ^68^Ga^3+^ in the complexation process with bifunctional chelators^29^. The concentrations determined for both ions were extremely low, always under 100 ppb (Table 2). A common impurity expected in the ^68^Ga eluate of commercial SnO_2_-based generators is the Sn ion due to the dissolution of the column matrix. It is of interest that Sn concentration was low, even under 100 ppb when the generator is operated in “dry” mode. Increased concentration of trace metals was not observed in the first elution relative to the second day or after (data not shown), which is something that has been described by other authors^10^. The elution was directly evaluated for radiolabelling of DOTA-TOC without performing any additional purification process due to the relatively low concentration of trace metals.

**Table 2:** Concentration of the metallic impurities of the ^68^Ge/^68^Ga generator in the ^68^Ga eluates.

Metal	Period	Concentration µg/L (ppb)	Metal	Period	Concentration µg/L (ppb)	Metal	Period	Concentration µg/L (ppb)
Ag	1	–	Co	1	0.47 ± 0.27 (n = 2)	Ni	1	13 ± 13 (n = 5)
	2	–		2	0.56 (n = 1)		2	12 ± 10 (n = 2)
	3	–		3	–		3	–
Al^a^	1	1.5 (n = 1)	Cr	1	79 ± 32 (n = 7)	Pb	1	178 ± 133 (n = 6)
	2	11 ± 6.5 (n = 3)		2	31 ± 26 (n = 8)		2	605 ± 1,021 (n = 7)
	3	–		3	14 (n = 1)		3	6,708 (n = 1)
As	1	2.9 (n = 1)	Cu	1	–	Sb	1	5.3 ± 3.5 (n = 6)
	2	2.4 ± 1.7 (n = 7)		2	36 ± 43 (n = 5)		2	12 ± 14 (n = 7)
	3	1.2 ± 0.4 (n = 3)		3	20 (n = 1)		3	164 (n = 1)
Ba	1	25 ± 24 (n = 7)	Fe	1	1.6 (n = 1)	Sn	1	254 ± 85 (n = 7)
	2	170 ± 80 (n = 8)		2	4.2 ± 3.3 (n = 8)		2	112 ± 48 (n = 8)
	3	112 ± 140 (n = 6)		3	–		3	44 ± 29 (n = 6)
Bi	1	–	In	1	1.3 ± 0.4 (n = 6)	Ti	1	5.0 ± 1.9 (n = 3)
	2	0.7 (n = 1)		2	0.78 ± 0.02 (n = 5)		2	17 ± 18 (n = 8)
	3	–		3	–		3	3.7 ± 4.1 (n = 3)
Ca	1	159 (n = 1)	Mg	1	1653 (n = 1)	Zn^a^	1	91.5 (n = 1)
	2	799 ± 774 (n = 8)		2	400 ± 373 (n = 6)		2	187 ± 125 (n = 8)
	3	173 (n = 1)		3	188 (n = 1)		3	6.4 ± 7.9 (n = 3)
Cd	1	–	Mn	1	30 ± 28 (n = 2)			
	2	0.8 ± 0.2 (n = 3)		2	119 ± 102 (n = 8)			
	3	–		3	–			

The metal ion content was determined in 21 elutions obtained from three different periods: (1) elutions from 24 to 61 (in 7 elutions; ^68^Ga eluted at 0.5 M HCl, “wet” mode), (2) elutions from 75 to 124 (in 8 elutions; ^68^Ga eluted at 1 M HCl, “wet” mode) and (3) elutions from 263 to 275 (in 6 elutions; ^68^Ga eluted at 1 M HCl, “dry” mode). The 1 M HCl was measured as the blank sample. Only are shown metal concentrations higher than the Limit of Detection (LD = 0.5 µg/L) and the blank sample (BS). The data are expressed as mean ± standard deviation, where *n* represents the number of elutions > LD and > BS.

^a^ Concentrations of Al and Zn are expressed in mg/L (ppm).

### Radiolabelling

^68^Ga was eluted from the generator operating in “wet” mode using 1 M HCl solution at flow rate of 1 mL/min. Radiolabelling of [^68^Ga]Ga-DOTA-TOC was carried out by straightforward one-step synthesis (see “Materials and methods”). The radiolabelling yield (RY) of [^68^Ga]Ga-DOTA-TOC was dependent on the amount of peptide used; thus, RY was 27.51 ± 14.93% for 2–4 µg (n = 20) and 60.37 ± 28.56% for 5–20 µg (n = 6) of DOTA-TOC. Although some elutions contained Zn impurities, the RY are in line with previous results using ^68^Ga eluates from SnO_2_ and TiO_2_ commercial generators without a post-elution purification procedure to label DOTA-peptides^13^, although slightly lower in comparison to those reported using other types of generators^13, 29^, such as CeO_2_-PAN and SiO_2_ (Supplementary Table S2, online). In addition, we prepared other ^68^Ga-complexes with high yields using ligands such as NODAGA-(RGD)_2_, PSMA-11 and iron-oxide nanoparticles (data not shown). Although some authors have replaced HEPES by sodium acetate as radiolabelling buffer (since this is an easily available and pharmacologically harmless buffer^30)^, we decided to use HEPES because it increases the radiolabelling yield and, although HEPES is currently not approved for human use, we believe that it is the most appropriate buffer for use in the preparation of ^68^Ga-DOTA-peptides for small animal PET imaging.

The eluate of ^68^Ge/^68^Ga generator is not chemically pure so non-radioactive metallic impurities may compete with ^68^Ga for coordinative labelling of radiopharmaceuticals precursors^31^. The metal ion content of the ^68^Ge/^68^Ga generator eluate depends on elution frequency. Therefore, a preventive elution 4 h prior to the synthesis has been recommended since the interfering metal ion concentration can be kept to a minimum^10^. We saw no differences between the first elution of the generator each day and an elution 3–4 h later with respect to labelling yield, which perhaps is due to the low amount of metal ion traces present in the eluate. Additionally, using an excess of precursor in the synthesis procedure could contribute to this situation.

**Figure 4:**
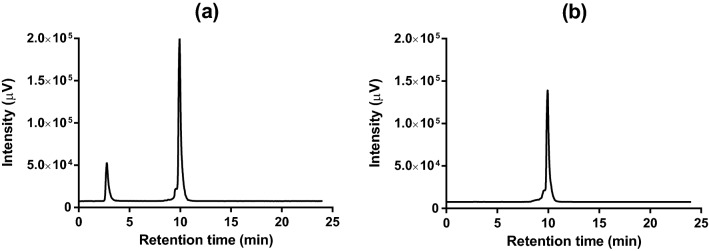
HPLC radiochromatogram of [^68^Ga]Ga-DOTA-TOC before (**a**) and after (**b**) purification step by Sep Pak. R_t_ (free ^68^Ga) = 2.78 min and R_t_ ([^68^Ga]Ga-DOTA-TOC) = 9.94 min.

Representative HPLC-radiochromatogram before and after purification from the C-18 Sep-Pak (Fig. 4a,b) showed a peak corresponding to [^68^Ga]Ga-DOTA-TOC with retention time of 9.94 min. Free ^68^Ga showed a retention time of 2.78 min (Fig. 4a). After Sep Pak purification, free ^68^Ga was removed and radiochemical purity (RQP) of the final product was determined to be ≥ 97%. An unresolved front shoulder before the mean peak could be observed in both chromatograms; this poorly separated shoulder was estimated to be < 3% of total radioactivity regardless of the amount of starting activity. These radioimpurities are partially attributable to the radiolytic oxidation of [^68^Ga]Ga-DOTA-TOC, which is consistent with previous results^26^. The addition of radical scavengers such as ascorbic acid could significantly suppress by-product formation^32^.

### PET imaging with [^68^Ga]Ga-DOTA-TOC in a preclinical mouse model of lung cancer

We used lung cancer mouse models generated in the laboratory to determine the suitability of the [^68^Ga]Ga-DOTA-TOC obtained for detection of tumors in vivo by PET imaging. We have used a high-grade neuroendocrine lung cancer animal model rendering Large-Cell Neuroendocrine Carcinoma (LCNEC)^33, 34^. LCNEC is an aggressive, poorly differentiated pulmonary neuroendocrine tumor which expresses a high density of surface somatostatin receptors (SSTR). This allows imaging with radiolabelled somatostatin analogue DOTA-TOC^35^.

**Figure 5:**
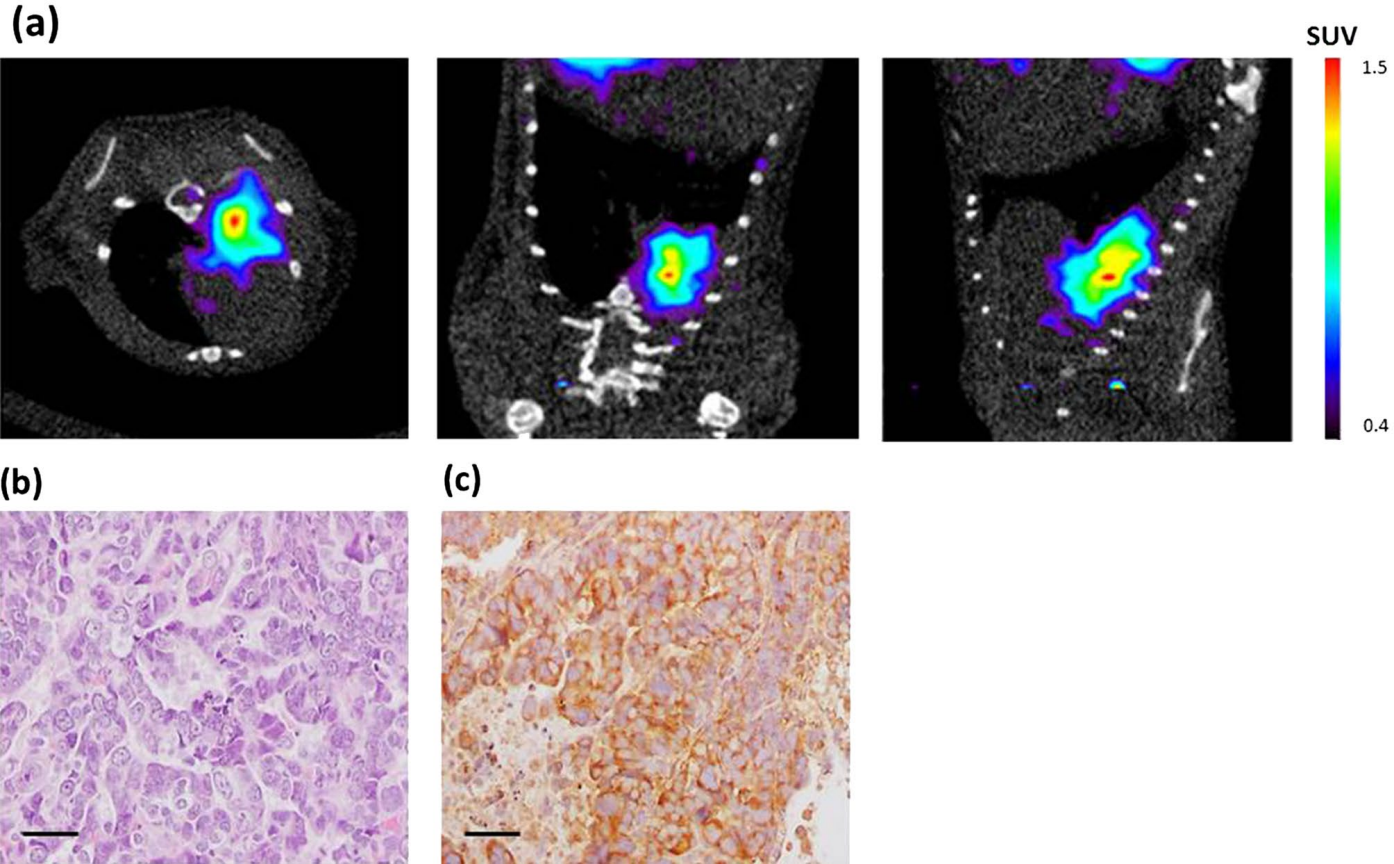
(**a**) Micro-PET/CT images of [^68^Ga]Ga-DOTA-TOC in a mouse model of LCNEC (axial, coronal and sagittal view). Radiolabelled DOTA-TOC accumulated in lung tumor 30 min post-injection. (**b**) Hematoxylin–eosin (H&E) stained lung tumor section at necropsy. (**c**) Immunohistochemical analyses of Somatostatin Receptor 2 (SSTR2) showing the expression of the protein in the tumor. Scale bars = 100 µm.

Micro-PET/CT [^68^Ga]Ga-DOTA-TOC showed increased uptake in some areas of the lungs of the cancer developing mouse models (Fig. 5a). No signal, or background signal, was detected in healthy control mice (not shown). Histological analyses allowed accurate lung cancer diagnosis which revealed Large-Cell Neuroendocrine Carcinoma in the lungs of the tested mice (n = 4) (Fig. 5b). Expression of Somatostatin Receptor 2 (SSTR2) in the tumors (Fig. 5c) was confirmed by immunohistochemistry. These studies demonstrate that early detection of these tumors is feasible due to their avidity for ^68^Ga-DOTATOC that allows follow-up and monitoring changes after therapy. Thus, small animal in vivo PET imaging tumor detection using the [^68^Ga]Ga-DOTA-TOC is a powerful tool in preclinical evaluation of cancer development with potential translational and therapeutic applications.

## Conclusions

Nano-crystalline SnO_2_ provided column support for a ^68^Ge/^68^Ga generator system which was prepared and evaluated over a period of more than 1 year of use and 300 elutions. The generator produced readily and reproducibly carrier-free ^68^Ga by elution with 1 M HCl solution. The elution yield of ^68^Ga was > 90% in the first 7 ml when the generator was new and subsequently decreased with intensive use to around 70%. ^68^Ge content in the eluate did not exceed 1 × 10^–4^%. Using either the “wet” or “dry” elution mode of the ^68^Ge/^68^Ga generator did not result in any noticeable differences in elution yield, therefore, the use of “dry” elution mode may be preferable for long-term applications in order to prevent tin oxide from being slightly attacked by the HCl used as eluent^36^. Furthermore, ^68^Ga direct elution from the generator, without the inclusion of a post-elution purification procedure, was successfully used for radiolabelling DOTA-TOC. The radiotracer [^68^Ga]Ga-DOTA-TOC showed a remarkable sensitivity for the in vivo detection of large-cell neuroendocrine tumors in a lung cancer mouse model.

## Materials and methods

### Preparation of ^68^Ge/^68^Ga radionuclide generator

The generator was prepared using a borosilicate glass column. The dimensions of the column were 100 mm in height and 6 mm of internal radius; filters in the form of porous frits (20 µm) were placed at the top and at the bottom of the column in order to avoid the presence of particulate impurities and to prevent disturbance of the column bed during flow of eluent solution through it. For the purpose of radioprotection, the column was kept in an easily-transportable lead shielded cylindrical shape (250 × 10 × 55 mm; height × inner radius × external radius) and all operations were carried out in a closed system using connecting Teflon tubes. A sterile polyethylene 10 mL syringe was attached to the inlet tube via a Luer-lock connection to elute ^68^Ga from the generator with HCl (Fig. 2a).

Excess of tin dioxide, synthesized on demand by Keeling & Walker (UK) and previously characterized by our research group^22^, was calcined at 900 °C for 3 h in an oven and cooled later to room temperature. Next, it was sifted through a 100–150 µm sieve in a stainless steel frame. A borosilicate glass column was packed with 4.5 g of nano-SnO_2_ and preconditioned with 50 mL of 0.5 M HCl to remove fine particles. Subsequently the column was loaded with 740 MBq of ^68^Ge in no-carrier-added form (obtained from JSC Isotope, Russia) and washed with 50 mL of 1 M HCl solution in order to remove trace levels of non-adsorbed ^68^Ge. One day after loading ^68^Ge, enough time for in-growth of the ^68^Ga activity, the generator was ready to use.

Gallium-68 was eluted from the generator using 7 mL of hydrochloric acid (0.5–1 M HCl) prepared from a solution of 34% hydrochloric acid, Ultrex II ultrapure reagent (J. T. Baker) at a constant flow rate of 1 or 1.5 mL/min provided by an infusion pump. The generator was designed to operate either in the “dry” or “wet” mode of operation. In the “dry” mode, after each elution the rest of HCl was removed by passing air through the column; it often happens that some HCl remains in the column despite drying. In the “wet” mode, after each elution the column remains filled with HCl till the next elution. The performance of the generator was evaluated for 17 months, eluting it regularly at intervals of once, twice or three times per day (more than 300 elutions during the period of study). The elution profile was studied by collecting the eluates in fractions of 0.5 mL and determining their activity. A dose calibrator model VDC-405 (Veenstra Instruments) was used for the determination of ^68^Ga activity after elution. The elution yield was expressed as the percentage of the eluted ^68^Ga activity in relation to the theoretical activity of ^68^Ga in the generator column after decay correction for the elapsed time. As the half-life of the ^68^Ge is much greater than the ^68^Ga, the theoretical activity of ^68^Ga was calculated by using the equation corresponding to secular radiochemical equilibrium:

$${\mathrm{A}}_{\mathrm{d}}\cong {A}_{p}\left(1-{e}^{{\lambda }_{d}t}\right)$$

where A_p_ and A_d_ are the activity of ^68^Ge and ^68^Ga respectively and λ_d_ (0.0102 min^−1^)^1^ is the radioactive decay constant of ^68^Ga.

The activity of ^68^Ge in aliquots of eluates was analyzed by gamma-ray spectrometry using a calibrated coaxial High Purity Germanium (HPGe) detector model GEM 13,200 (Ortec) coupled to multichannel gamma-ray analyzer DSA1000 (Canberra). The generator eluates were analyzed after at least 48 h in order to allow the ^68^Ga to decay to a level that permits the detection of ^68^Ge since it can be detected indirectly as a decay product, ^68^Ga. All samples, which contained 1 mL, were measured using the same conditions: constant geometry using 1.5 mL conical bottom tubes made from polypropylene, placed at a distance of 5 cm from the detector and 6 h as minimum measurement time. The activity of each sample was calculated by quantifying the area of 511 keV peak in the spectrum obtained, which corresponds to radioactive decay of ^68^Ga. The Minimum Detectable Activity (MDA) for ^68^Ga was 1 Bq. Finally, the ^68^Ge activity was calculated by direct comparison with the results obtained from the measurement of a standard source of ^68^Ge, performed by Ionizing Radiations Metrology Laboratory belonging to the CIEMAT, under the same measurement conditions previously mentioned. The generator column was visually checked additionally by small animal PET/CT SuperArgus (SEDECAL, Spain) with the purpose of studying the possible spread of ^68^Ge zone along the column due to the number of elutions and the time of use of the generator.

The presence of potential chemical impurities (in the form of Ag, Al, As, Ba, Bi, Ca, Cd, Co, Cr, Cu, Fe, In, Mg, Mn, Ni, Pb, Sb, Sn, Ti and Zn) in the ^68^Ga eluate was determined in three groups according to the elution number: 24–61, 75–124 and 263–275. The trace level detection of the metal ion contamination in decayed samples were carried out by Inductively Coupled Plasma Mass Spectrometry (ICP-MS) using a quadrupole single collector iCAP Q (Thermo Scientific) equipped with a collision cell. The water used was Milli-Q purity (Millipore) and the acids were purified by Sub-Boiling distillation using a duoPUR system (Milestone). The non-disposable materials used were previously washed with 5% HNO_3_ and distilled water. Quantification was carried out by external calibration and internal standard, preparing the standards by successive dilution of a certified standard (Alfa Aesar and Inorganic Ventures) to 2–5% (v/v) using 65% HNO_3_ (Sigma-Aldrich) for each of the elements.

### Radiolabelling

Labelling properties of the ^68^Ge/^68^Ga generator were tested by a method based on the radiolabelling of DOTA-TOC (DOTA-[Tyr3]-octreotide), provided by ABX (Radeberg, Germany). This peptide, a DOTA derivatized somatostatin analog, shows high affinity to the SSTR2 subtype of somatostatin receptor expressing tumors and binds the trivalent Ga^3+^ with a high kinetic and thermodynamic stability. It is widely used in clinical routines for PET imaging of neuroendocrine tumors^37^. The procedure used to label, purify and determine the radiochemical purity of [^68^Ga]Ga-DOTA-TOC was essentially the same as that used by our group^33^ with some adjustments. A solution of 210 ± 5 mg HEPES (Sigma-Aldrich) dissolved in 1 mL of deionized water was transferred into a reaction vial which contained the peptide DOTA-TOC previously dissolved in water. The ^68^Ge/^68^Ga-generator was eluted with 7 ml of 1 M HCl solution (prepared from 34% HCl, Ultrex II ultrapure reagent, J. T. Baker) and 1 mL of the eluate was transferred to the reaction vial. The final pH of the reaction mixtu re was 3.5–4, measured by indicator paper strips (Merck). After very careful shaking, the mixture was heated for 5 min at 90 ± 5 ºC using a microwave with monomodal radiation and after cooled to room temperature with nitrogen.

The crude product was purified by solid-phase extraction cartridge Sep Pak light C18 cartridge (Waters) previously conditioned and equilibrated with 4 mL of pure ethanol and 4 mL of deionized water. The mixture was transferred onto Sep Pak where [^68^Ga]Ga-DOTA-TOC and colloidal ^68^Ga were retained, whereas free ^68^Ga passed through the cartridge. Next, the cartridge was rinsed with 4 mL of deionized water and the activity on the Sep Pak cartridge was reversely recovered with 0.5 mL ethanol 96% (v/v) (Scharlab); during this procedure all colloidal ^68^Ga was retained on the Sep Pak cartridge. Finally, ethanol was evaporated to dryness using a Speed Vac (Savant) and [^68^Ga]Ga-DOTA-TOC was dissolved with a 0.9% NaCl solution (Braun). The radiolabelling yield was calculated by comparing the fraction of ^68^Ga collected in ethanol with the activity in the initial radiolabelling reaction using decay-corrected radioactivity values.

The radiochemical purity of [^68^Ga]Ga-DOTA-TOC was determined by radio-High Performance Liquid Chromatography (radio-HPLC) (Jasco) equipped with a photo diode array detector MD-4015 (Jasco), a radioactivity detector LB 507A (Berthold) and the measurements performed under the following conditions. Column: Jupiter Proteo 90 Å column (Phenomenex), 4 µm, 250 × 4.6 mm. Eluents: 0.1% trifluoroacetic acid (TFA) in 5% acetonitrile (solvent A) and 0.1% TFA in 95% acetonitrile (solvent B). Gradient: 0–2 min (5% B), 2–17 min (5–100% B), 17–20 min (100% B), 20–22 min (100–5% B) and 22–24 min (5% B). Flow rate: 1 mL/min. Two aliquots, before and after purification step by Sep Pak, were taken to determine radiochemical purity. TFA was purchased from Sigma-Aldrich and Acetonitrile Ultra Gradient HPLC grade from J. T. Baker.

### Preclinical model for pulmonary neuroendocrine tumors

All animal experiments were approved by the Animal Ethical Committee of the Centro de Investigaciones Energéticas, Medioambientales y Tecnológicas (CIEMAT) and conducted in compliance with institutional and national guidelines. A mouse model of high-grade neuroendocrine lung cancer (LCNEC) based on the ablation of four tumor suppressor genes (Rb, Rbl1,pTEN and Trp53) was used to perform imaging studies^33, 34, 38^.

### PET/CT imaging

PET studies were performed in an Argus PET/CT (SEDECAL) scanner. The procedure used was as described by our group^33^. Briefly, PET studies (energy window 250–700 keV and 30 min static acquisition) and CT studies (voltage 45 kV, current 150  μA, 8 shots, 360 projections, and standard resolution) were performed at 90 min after intravenous injection of [^68^Ga]Ga-DOTA-TOC (2–5 MBq) in mice anesthetized by inhalation of 2–2.5% of 2–2.5% isoflurane (Esteve) in 100% oxygen (ALPHAGAZ™, Air Liquide) at a flow rate of 5% oxygen using a Fluovac System (Harvard Bioscience). PET images were corrected for random events and scatter with and without attenuation correction and reconstructed using the 2D–OSEM (Ordered Subset Expectation Maximization) algorithm (16 subsets and 2 iterations). For image-reading purposes PET images were fused with the corresponding CT images, which were used as a reference for image co-registration. Images were analyzed using the image analysis software AMIDE, version 1.0.4 (https://amide.sourceforge.net).

### Histology and Immunohistochemistry

Histology and Immunohistochemistry tests were performed to confirm the diagnosis of the tumor and the expression and localization of SSTR2. Lungs were perfused at necropsy with 4% formaldehyde, fixed and embedded in paraffin wax sections (5 μm) were stained with hematoxylin and eosin (H&E) for histological analysis or processed for immunohistochemistry, essentially as in previously described standard protocols^39^. For expression of SSTR2, a high temperature antigen unmasking technique consisting of 15-min microwaving of slides in 0.01 M citrate buffer (Antigen Retrieval Buffer, Abcam) was used after deparaffinization to enhance the staining. Sections were then incubated with 5% horse serum (Gibco) for 30 min to block the Fc receptor in tissue, and then washed three times with sterile PBS (Gibco, pH 7.5) prior to incubation with the anti-SSTR2 primary antibody (Sigma-Aldrich, diluted 1:100). Peroxidase-conjugated secondary antibody supplied by the manufacturer was used at 1:300 dilution and visualized using diaminobenzidine as a substrate (DAB kit Vector). Control slides were obtained by replacing primary antibodies with PBS (data not shown).

### Statistical analyses

Continuous measurements are presented as mean ± standard deviation (μ ± σ), unless otherwise stated. Mean values were compared using one-way ANOVA followed by post hoc testing or custom contrasts. The difference was considered statistically significant when *p* value was < 0.05. Statistical analyses were performed using SPSS in 14.0.

## Supplementary information

Supplementary Information

## Data Availability

The datasets generated and analyzed during the current study are available from the corresponding author upon reasonable request.
