# Author Correction: Development and long-term evaluation of a new ^68^Ge/^68^Ga generator based on nano-SnO_2_ for PET imaging

**DOI:** 10.1038/s41598-020-76492-6

**Published:** 2020-11-18

**Authors:** Eduardo Romero, Alfonso Martínez, Marta Oteo, Marta Ibañez, Mirentxu Santos, Miguel Ángel Morcillo

**Affiliations:** 1 Biomedical Applications and Pharmacokinetics Unit, CIEMAT, 28040 Madrid, Spain; 2 Molecular Oncology Unit, CIEMAT, 28040 Madrid, Spain

Correction to: *Scientific Reports* 10.1038/s41598-020-69659-8, published online 29 July 2020

This Article contains errors in Table 1.

In the column, “Company”,

“IRE EliT (Galli Eo®)”

should read:

“IRE EliT (Galli Eo®)(Galli Ad®)”

In the column, “Column matrix”,

“Unspecified”

should read:

“TiO_2_”

In the column, “Initial ^68^Ga elution yield (%)”,

“ > 65”

should read:

“ > 80”

As a result, in the Introduction,

“Another emerging generator is Galli Eo (from IRE EliT, Fleurus, Belgium), however, the column contains an unspecified resin. The generator is eluted with 0.1 M HCl, and has more than 67% elution yield and less than 1 × 10^–3^% ^68^Ge breakthrough^18^. According to its brochure, metal content per elution is less than 1 ppm and ≤ 10 µg/GBq of ^68^Ga for Fe, Cu, Ni, Zn, Pb and Al. The Eckert & Ziegler GalliaPharm® and the IRE ELiT Galli Eo® generators are both GMP grade and have type II drug master files on file with the FDA.”

should read:

“Another emerging generator is Galli Eo (from IRE EliT, Fleurus, Belgium) which is based on TiO_2_. The generator is eluted with 0.1 M HCl, and has more than 67% elution yield and less than 1 × 10^–3^% ^68^Ge breakthrough^18^. According to its brochure, metal content per elution is less than 1 ppm and ≤ 10 µg/GBq of ^68^Ga for Fe, Cu, Ni, Zn, Pb and Al. The Eckert & Ziegler GalliaPharm® and the IRE ELiT Galli Eo® generators are both GMP grade and have type II drug master files on file with the FDA. Recently, the IRE ELiT generator has been authorized in several European countries (Galli Ad®) and its initial elution yield has been improved (> 80%, personal communication from IRE ELiT company).”

