# Supplementary material for: Development and long-term evaluation of a new 68Ge/68Ga generator based on nano-SnO2 for PET imaging
Source: Sci Rep. 2020 Jul 29;10:12756. doi: 10.1038/s41598-020-69659-8 (PMC7392752; doi:10.1038/s41598-020-69659-8)
Supplement: Supplementary file 1 — Supplementary Information [file 41598_2020_69659_MOESM1_ESM.docx]

**Supplementary Information**

**to**

**Development and long-term evaluation of a new ^68^Ge/^68^Ga generator based on nano-SnO_2_ for PET imaging**

**Eduardo Romero^1^, Alfonso Martínez^1^, Marta Oteo^1^, Marta Ibañez^1^, Mirentxu Santos^2^, Miguel Ángel Morcillo^1,*^**

^1^Biomedical Applications and Pharmacokinetics Unit, CIEMAT, Madrid, E-28040, Spain

^2^Molecular Oncology Unit, CIEMAT, Madrid, E-28040, Spain

Content:

Page 2: Elution yield of ^68^Ga and ^68^Ge breakthrough for the generator over the experimental period.

Page 4: Radiolabelling yields of DOTA-peptides/NCS with ^68^Ga

Table S1 – ^68^Ga elution yield and ^68^Ge breakthrough for the nano-SnO_2_ ^68^Ge/^68^Ga generator over 17 months (305 elutions). Data are expressed as mean±standard deviation and 95% confidence interval of mean, unless otherwise stated, where *n* represents the number of elutions evaluated.

| **Elutions** | 1-68 | 69-118 | 119-159 | 160-218 | 219-233 | 234-253 | 254-278 | 279-305 |
| --- | --- | --- | --- | --- | --- | --- | --- | --- |
| **Operation:**  **Mode**  **HCl (M)**  **Flow rate (ml/min)** | Wet  0.5  1 | Wet  1  1 | Wet  1  1.5 | Wet  1  1 | Dry  1  1 | Wet  1  1 | Dry  1  1 | Wet  1  1 |
| **^68^Ga elution yield (%)** | 70.1±3.5  (69.2-70.9)  n=68 | 91.1±1.8  (90.6-91.6)  n=50 | 86.3±2.1  (85.7-87.0)  n=41 | 83.5±1.5  (83.1-83.9)  n=59 | 80.1±3.8  (78.0-82.2)  n=15 | 81.4±2.9  (80.1-82.8)  n=20 | 77.9±3.4  (76.5-79.3)  n=25 | 73.8±1.9  (73.1-74.7)  n=27 |
| **^68^Ge breakthrough (%)**  (x 10^-5^) | 6.0±3.1  (5.0-6.9)  n=44 | 5.6±1.5  (5.0-6.2)  n=27 | 5.7±1.7  (4.9-6.4)  n=23 | 4.8±0.6  (4.5-5.1)  n=21 | 4.6±0.2  (4.3-4.9)  n=5 | 4.8±0.6  (4.4-5.3)  n=8 | 5.3±1.0  (4.4-6.1)  n=8 | 5.0±0.5  (4.6-5.3)  n=9 |

A one-way Welch ANOVA followed by custom contrasts was conducted to determine whether there was a difference between two groups or a combination of more than two groups of elutions. There was a statistically significant increase in ^68^Ga elution yield from the group of elutions with 0.5M HCl (70.1±3.5%) to group with 1M HCl (91.1±1.8%), with a mean increase of 21.0±0.5% [mean ± standard error], p < .0005. The differences between groups of elutions done in dry and wet mode was not statistically significant, p < .058. With regard to the ^68^Ge breakthrough, no statistically significant differences were found between these groups of elutions.

A one-way Welch ANOVA followed by post hoc testing was conducted to determine if the ^68^Ga elution yield with 1M HCl operated in wet mode (1 ml/min) changed over time. The ^68^Ga elution yield was statistically significantly different between different groups of elutions, Welch´s *F*(3, 503.544) = 502.546, p < .0005. Games-Howell post hoc analysis revealed that the decrease of ^68^Ga elution yield from 69-118 to 160-218 elutions (7.6%, 95% CI (6.8 to 8.5%)) was statistically significant (p < .0005), as well as the decrease from 69-118 to 234-253 elutions (9.7%, 95% CI (7.7 to 11.6%), p < .0005) and from 69-118 to 279-305 elutions (17.3%, 95% CI (16.1 to 18.5%), p < .0005).

A one-way Welch ANOVA followed by post hoc testing was also conducted to determine if the ^68^Ge breakthrough after elution with 1M HCl operated in wet mode (1 ml/min) changed with the number of elutions. ^68^Ge breakthrough was not statistically significantly different between different groups of elutions, Welch´s *F*(3, 2.138) = 2.736, p = .051. The mean value was 0.000052±0.000011% (n=65).

Table S2 – Comparison of radiolabelling yields of DPTA-peptides or DOTA-NCS with ^68^Ga eluted from different ^68^Ge/^68^Ga generators. Values are shown as mean±standard deviation.

| **Generator** | **Compound** | **Radiolabelling yield (%)** |
| --- | --- | --- |
| nano-SnO_2_ (this work) | DOTA-TOC (5-20 µg) | 60.4 ± 28.6 |
| SnO_2_ (iThemba) | DOTA-RGD_2_ (20 µg) | 36.8 ± 2.8^a^ |
| TiO_2_ (Eckert & Ziegler) | DOTA-RGD_2_ (20 µg) | 67.2 ± 3.2^a^ |
| SiO_2_ (ITG) | DOTA-RGD_2_ (20 µg) | 97.6 ± 2.7^a^ |
| CeO_2_-PAN (in-house) | DOTA-RGD_2_ (20 µg)  DOTA-NCS (10 ug)  DOTA-NCS (20 µg) | 96.6 ± 1.8^a^  48 ± 4^b^  99 ± 1^b^ |

Data taken from references [13]^a^ and [29]^b^
